# Relationship between IDH1/2 and TERT promoter mutation and the prognosis of human glioma patients

**DOI:** 10.12669/pjms.39.3.7149

**Published:** 2023

**Authors:** Xipeng Liu, Ming Liu, Bing Cao, Jianxin Qiao, Xiufeng Zhang

**Affiliations:** 1 Xiping Liu, Department of Neurosurgery, The First Affiliated Hospital of Hebei North University Zhangjiakou 075000, Hebei Province, P.R. China; 2 Ming Liu, Department of Neurosurgery, The First Affiliated Hospital of Hebei North University Zhangjiakou 075000, Hebei Province, P.R. China; 3 Bing Caom, Department of Neurosurgery, The First Affiliated Hospital of Hebei North University Zhangjiakou 075000, Hebei Province, P.R. China; 4 Jianxin Qiao, Department of Neurosurgery, The First Affiliated Hospital of Hebei North University Zhangjiakou 075000, Hebei Province, P.R. China; 5 Xiufeng Zhang, Department of Neurosurgery, The First Affiliated Hospital of Hebei North University Zhangjiakou 075000, Hebei Province, P.R. China

**Keywords:** Human glioma, Isocitrate dehydrogenase, Telomerase reverse transcriptase, Promoter region mutation

## Abstract

**Objective:**

To investigate the relationship between isocitrate dehydrogenase (IDH) 1/2 mutation, telomerase reverse transcriptase (TERT) gene promoter mutation and the prognosis of human glioma patients.

**Methods:**

One hundred fifteen patients with human glioma, treated surgically in The First Affiliated Hospital of Hebei North University from January 2019 to January 2020, were included. All patients were followed up until January 31, 2022. The mutations of IDH1/2 and TERT promoter were analyzed, and risk factors affecting survival of the patients with glioma were assessed.

**Results:**

IDH1 gene mutation occurred in 82 cases, IDH2 gene mutation occurred in five cases and TERT promoter mutation occurred in 54 cases. Univariate analysis showed that tumor WHO grade, resection range, preoperative Karnofsky performance status score, postoperative radiotherapy and chemotherapy, IDH1/2 gene and TERT promoter mutation influenced postoperative survival of patients with glioma (P<0.05). Kaplan-Meier survival curve showed that IDH1/2 gene and TERT promoter mutation were significantly different from those of wild-type patients (P<0.05).

**Conclusion:**

IDH1/2 gene and TERT promoter mutations are more frequent in patients with human glioma. These related factors can be used as molecular markers to aid in the prognosis of patients with glioma.

## INTRODUCTION

Glioma is the most common malignant tumor found in the central nervous system, accounting for ~70% of primary central nervous system tumors, with an incidence rate increasing year by year.^1^ Presently, the clinical treatment of glioma patients is focused on the combined treatment of surgery, radiotherapy and chemotherapy. Although this protocol can improve patient survival, the methodology is not ideal. Specifically, the unclear tumor boundary, difficulty in complete resection, recurrence, and poor prognosis, these are the urgent problems to be solved in clinical medicine.^2^ Improved medical molecular biotechnology has gradually increased clinical research on tumor biological characteristics. Molecular pathological detection can better evaluate the degree of malignancy and prognosis of tumors.^3^

Notably, isocitrate dehydrogenase (IDH) gene mutation can modify cell metabolism and may be involved in the occurrence and development of tumors.^4^ IDH1/2 gene mutation is the most common metabolic enzyme gene mutation, is more common in some subtypes of acute myeloid leukemia and glioma, and is related to the occurrence, development and prognosis of glioma.^5^,^6^ Telomerase reverse transcriptase (TERT) promoter region mutation is a point mutation in the core sequence of the TERT promoter region. TERT occurs widely in a variety of malignant tumors, is related to the prognosis of patients,^7^ and is highly expressed in glioma tissues.^8^ In this study, the gene mutation of IDH1/2 and TERT promoter region was sequenced, and the postoperative follow-up was carried out. The relationship between the gene mutation and the occurrence, development and prognosis of glioma, was examined to provide a theoretical basis, and possible therapeutic target, for the treatment of glioma.

## METHODS

The clinical records of 115 patients with human glioma who underwent surgery in The First Affiliated Hospital of Hebei North University from January 2019 to January 2020 were collected. There were 63 males and 52 females, aged 36-71 years, with an average age of 54.4±9.4yrs. Tumor diameter ranged from 0.9~7.8cm, with an average of 4.8±1.6cm. WHO grading of tumor found 18 cases of Grade-II, 40 cases of Grade-III and 57 cases of Grade-IV.^9^ Total resection was performed in 52 cases and partial resection in 63 cases, while the KPS score found 31 cases <60 points, 84 cases ≥60 points.^10^ This study has been approved by the ethics committee of First Affiliated Hospital of Hebei North University (Approval No.: K2022067, Date: 2022-05-16).

### Inclusion criteria:

- The glioma diagnosis was confirmed by pathological examination;
- All were first visits;
- All tumors were single focus;
- The pathological data and clinical data are complete.

### Exclusion criteria:

- Combined with tumors in other parts;
- Radiotherapy and chemotherapy were performed before operation;
- Patients with surgical contraindications or cognitive impairment.

Tumor tissue samples were obtained by surgical resection before radiotherapy and chemotherapy. The resected samples were quickly frozen and stored in liquid nitrogen for subsequent DNA extraction and paraffin embedding. Qiaamp DNA Mini Kit (Manufacturer: Qiagen, Germany) was used. The experimental steps were carried out in accordance with the instructions of the kit. The extracted products were quantified by NanoDrop Nd-1000 UV spectrophotometer (Manufacturer: NanoDrop company, USA), and the DNA integrity was detected by 1% agarose gel electrophoresis. The DNA samples were stored at -80^0^C for further analysis.

DH1 R132 locus and IDH2 R172 locus were amplified by PCR, the forward and reverse primers of IDH1 were GCTTTCGAGGGGTTGGAAAAGTC and Biotin-TTGCAAACATCACTGTTTGATC, respectively. IDH2 forward and reverse primers are ATCTTGGCGGTGACTTTCGC and biotin, respectively. The PCR products were further purified to obtain single stranded DNA, mixed with 40μL sequencing Buffer, denatured at 80^0^C for 2min, and sequenced and analyzed by Pyromark ID sequencer (Assisted by the joint laboratory of Luoxi Medical Technology Co., Ltd. and Gene Detection Technology Application Demonstration Center).

Nested PCR primers were designed to make the amplified sequence include TERT promoter region chr5 (1295250 locus and 1295228 locus). The forward and reverse primers of the first round of PCR were: 5’-GTCTAGCCAGTTCCTCCT-3’, 5’-GCTCCTACCGGGAGCGG-3’. The forward and reverse primers of the second round of PCR were: 5’-TCTCGATCCCGGACCCT-3’, 5’-GGACTTTAGGTTACAGG-3’. The total volume of PCR was 50μL, including 5μL template DNA (about 250ng), 0.25μL Taq DNA polymerase (Takara company), and 5μL 10×PCR buffer, 4μL dNTPs (2.5mmol/L), 1μL positive and negative primers (20 μMol/L), ddH_2_O added to 50μL. The thermal cycle parameters were: pre denaturation at 95^0^C for 5min, followed by 35 cycles: denaturation at 95^0^C for 30s, annealing at 56^0^C for 40s, extension at 72^0^C for 50s, and total extension at 72^0^C for 10min. PCR products were analyzed by agarose gel electrophoresis and observed by gel imaging system (Assisted by the joint laboratory of Luoxi Medical Technology Co., Ltd. and Gene Detection Technology Application Demonstration Center).

All cases were followed up by clinic and telephone. The enhanced MRI of the head was rechecked every three months and every 1/2 year from the second year. The overall survival time was from glioma on the day of operation to death or the end of follow-up. Lesion progression and recurrence was determined through assessment of the patient’s symptoms and imaging results. The follow-up period ended on January 31, 2022. Any patients who died after operation and of non-primary disease after operation and those who were lost to follow up were excluded.

Data was analyzed using SPSS 24.0 software. Measurement data is expressed in (*χ̅*±*S*), and a t-test was used to compare outcomes. The counting data is expressed by rate (%), which is expressed by *χ^2^*. While survival analysis was performed by Log-rank test. The Cox proportional hazard model was used for single factor and multi factor analysis. When *P*<0.05, the difference was statistically significant.

## RESULTS

A total of 115 glioma patients were included, of which 82 (71.3%) patients expressed IDH1 gene mutation and five (4.35%) patients expressed IDH2 gene mutation. There was no simultaneous mutation of IDH1 and IDH2 gene in the same sample. TERT promoter mutation was detected in 54 patients (50%). Gene mutation of glioma patients with different WHO grades is shown in Table-I.

**Table-I:** Gene mutations in patients with different grades of gliomas.

WHO classification	IDH1 mutation rate	IDH2 mutation rate	TERT promoter mutation rate
II	94.4% (17/18)	0.0% (0/18)	27.8% (5/18)
III	75.0% (30/40)	7.5% (3/40)	55.0% (22/40)
IV	61.4% (35/57)	3.5% (2/57)	47.4% (27/57)

**Fig.1:**
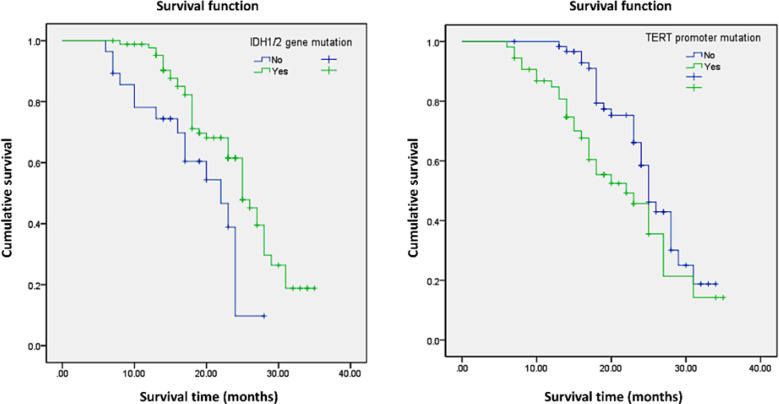
Survival curve of IDH1/2 and TERT promoter mutation and wild-type patients.

Univariate analysis showed that tumor WHO grade, resection range, preoperative KPS score, postoperative radiotherapy and chemotherapy, IDH1/2 gene mutation and TERT promoter mutation significantly affected the postoperative survival of patients with glioma (*P*<0.05), as shown in Table-II. The survival curve showed that there was a significant difference in survival between IDH1/2 gene and TERT promoter mutation patients and wild-type patients (*P*<0.05), as shown in Fig.1.

**Table-II:** COX univariate analysis of postoperative survival in patients with glioma

Factor	Classification	n=115	Mean survival time (months)	t/F/ χ^2^	p
Gender	Male	63	20.4±7.3	1.226	0.223
	Female	52	18.9±5.7		
Age (years)	<50	47	20.4±6.4	0.943	0.346
	≥50	68	19.2±6.7		
BMI (kg/m^2^)	≥22.5	60	18.6±6.6	1.719	0.088
	<22.5	55	20.7±6.5		
Smoking history	Yes	59	20.0±6.2	0.425	0.672
	No	56	19.5±7.0		
Drinking history	Yes	62	19.9±6.0	0.774	0.288
	No	53	19.5±7.1		
Tumor site	Lobe of brain	91	19.6±7.1	0.090	0.914
	ventricle	13	20.1±4.7		
	Hippocampal region	11	20.4±4.5		
WHO grading of tumor	Low level (II grade)	18	23.8±5.3	2.985	0.003
	High level (III~IV grade)	97	18.9±6.5		
Tumor resection range	Total cut	52	21.6±7.05	2.803	0.006
	Partial cut	63	18.2±5.8		
Preoperative KPS score	≥60	84	21.1±6.4	3.512	0.001
	<60	31	16.3±5.9		
Postoperative radiotherapy/chemotherapy	Yes	70	21.0±7.4	2.991	0.003
	No	45	17.7±4.6		
IDH1/2 mutation	Yes	87	20.8±6.4	3.183	0.002
	No	28	16.4±6.2		
TERT promoter mutation	Yes	54	17.4±6.9	3.721	<0.001
	No	61	21.8±5.6		

A Cox proportional multivariate analysis was conducted on the number of factors significantly affected by the above single factor analysis. Total tumor resection, preoperative KPS score ≥60, IDH1/2 gene mutation, postoperative radiotherapy and chemotherapy were associated with better survival of patients with gliomas, while TERT promoter mutation was associated with poor survival (*P*<0.05). Table-III.

**Table-III:** Cox multivariate analysis of postoperative survival of patients with glioma.

Factor	β	SE	Wald	HR	95%CI	p
Total tumor resection	-0.563	0.283	3.945	0.570	0.327~0.993	0.047
Preoperative KPS score ≥60	-0.839	0.295	8.092	0.432	0.242~0.770	0.004
Postoperative radiotherapy/chemotherapy	-0.608	0.295	4.251	0.544	0.305~0.970	0.039
IDH1/2 mutation	-0.651	0.315	4.265	0.522	0.281~0.967	0.039
TERT promoter mutation	0.572	0.273	4.393	1.771	1.038~3.024	0.036

## DISCUSSION

In this study, IDH1 gene mutation was detected in 86 cases (74.8%), IDH2 gene mutation in five cases (4.4%) and TERT promoter mutation in 68 cases (59.1%), the results showed that IDH1/2 and TERT genes were related factors affecting the prognosis of patients with glioma. Maimaiti A *et al*^11^ reported that the abnormal expression of many tumor related genes, such as the activation of oncogene and the inactivation of tumor suppressor gene, can lead to the occurrence of glioma, and the malignant degree of glioma and the prognosis of disease are closely related to the mutation, amplification or loss of certain specific genes. The meta-analysis results of Cui QK *et al*.^12^ showed that the ERCC2 rs13181 GT and TT genotypes were significantly associated with increased risk of glioma in Chinese population, with ORs(95%CI) of 1.47(1.17-1.85) and 1.50(1.02-2.22), but ERCC1 rs3212986 and ERCC2 rs13181 polymorphisms had no significant association with glioma risk in Caucasian populations.

However, Zhang L *et al*^13^ meta study failed to suggest that XRCC1 Arg280His polymorphism is associated with glioma risk. IDH catalyzes the decarboxylation of isocitrate to produce α-ketoglutarate. IDH1 is involved in the process of cell metabolism.^14^ Studies show that IDH1/2 gene mutation is an early event in the occurrence and development of glioma, 60-90% of IDH1 gene mutations occur in low-grade glioma.^14^,^15^ Watanabe *et al*^16^ analyzed glioma samples from patients with TP53 germline mutation syndrome and found that IDH1 mutation was R132C substitution, indicating that IDH mutation may precede TP53 mutation in the development process of glioma. Further, Juratli *et al*.^17^ showed that IDH1 and IDH2 gene mutations were mutually exclusive and did not exist in the same glioma sample. TERT is a kind of reverse transcriptase, which uses its own DNA molecules as a template to increase nucleotides at telomeres, while tumors can activate telomerase to maintain telomere length.^18^ The mutation in the core region of TERT promoter in glioma increases telomerase activity, indicating that TERT promoter mutation is related to the occurrence and development of glioma. Terzi *et al*^19^ showed that mutations in TERT promoter region accounted for about 50~60% of gliomas, and their histopathological types and proportions were different.

The univariate results showed that tumor grade, surgical resection range, preoperative KPS score, postoperative radiotherapy and chemotherapy, IDH1/2 gene and TERT promoter mutation were the influencing factors of postoperative survival of patients with glioma. Kaplan-Meier survival curve showed that IDH1/2 gene and TERT promoter mutation were significantly different from those of wild-type patients, again, it is suggested that IDH1/2 gene and TERT promoter mutations are related to the survival of patients with glioma. Ogura *et al*^20^ showed that IDH1 mutation caused the abnormal rise of 2-hydroxyglutaric acid and inhibited the activity of demethylase. Its mutation was positively correlated with P53 mutation and other states, which had a certain impact on the degree of tumor malignancy and drug sensitivity and could prolong patient survival time.

TERT promoter mutation can cause telomerase overexpression in tumor tissues or cells, which can promote carcinogenesis. TERT promoter mutation can affect the NF-κB signaling pathway which plays a regulatory role leading to the release of inflammatory mediators and aggravating tumor progression, resulting in further damage to the body, possibly increasing the risk of death.^21^ In addition, Cox multivariate analysis showed that total tumor resection, preoperative KPS score ≥60, postoperative radiotherapy and chemotherapy, and IDH1/2 gene mutation were associated with better postoperative survival of patients with glioma, while TERT promoter mutation was associated with poor postoperative survival. Highlighting that IDH1/2 gene and TERT promoter mutation are related to the prognosis of patients with glioma. By analyzing the factors that affect the survival of patients with brain glioma after surgery, this study concluded that mutations in IDH1/2 gene and TERT promoter have an impact on the prognosis of patients, but the specific mechanism by which they have a good or bad effect on the prognosis of patients is not yet very clear, just to guess that it may be related to the body’s antioxidant stress, and lay a certain foundation for further research on the mechanism.

### Limitations:

The sample size was small, and the relationship between IDH1/2 and TERT promoter and other molecular pathological indexes were not studied. It needs further in-depth research to explore the potential of treating glioma with both as targets.

## CONCLUSION

IDH1/2 gene and TERT promoter mutations are more frequent in patients with glioma. These mutations can be used as molecular markers to predict the prognosis of patients with glioma. Specifically, IDH1/2 gene mutation is associate with better prognosis, while TERT promoter mutation is associated with a poor prognosis.

### Authors’ Contributions:

**XL** conceived and designed the study.

**ML, BC**, **JQ** and **XZ** collected the data and performed the analysis.

**XL** was involved in the writing of the manuscript and is responsible for the integrity of the study.

All authors have read and approved the final manuscript.
